# Expression of transporter genes in anthelmintic resistant isolates of *Haemonchus contortus*

**DOI:** 10.1590/1678-4685-GMB-2023-0350

**Published:** 2024-08-16

**Authors:** Janaelia Ferreira Vasconcelos Rodrigues, Jessica Maria Leite dos Santos, Gracielle Araújo Frota, Luiz da Silva Vieira, Marcel Teixeira, Magaly Sales Monteiro, Jomar Patrício Monteiro

**Affiliations:** 1 Centro universitário INTA (UNINTA), Programa de Pós-Graduação em Biotecnologia, Sobral, CE, Brazil.; 2 Universidade Estadual Vale do Acaraú (UVA), Programa de Pós-Graduação em Zootecnia, Sobral, CE, Brazil.; 3 Embrapa Caprinos e Ovinos, Sobral, CE, Brazil.

**Keywords:** Nematode, benzimidazoles, macrocycle lactones, P-glycoproteins, resistance mechanisms

## Abstract

ATP-binding cassette (ABC) transporters, including P-glycoproteins (PGP), have been implicated in drug resistance in different organisms including *Haemonchus contortus*. This study confirmed the resistance status of *H. contortus* isolates selected for ivermectin (IVM) and oxfendazole (OXF) resistances using the fecal egg count reduction test and evaluated the gene expression of seven ABC transporters using RT-qPCR for two biological scenarios: the effect of selection for anthelmintic resistance and the effect of drug exposure on gene expression. Gene expression results showed that selection for IVM resistance led to the significant upregulation of *Hco-pgp-9a* (1.5-fold), *Hco-pgp-11* (3-fold) and *Hco-haf-9* (1.5-fold) (*p* < 0.05). Similarly, selection for OXF resistance led to the significant upregulation of *Hco-pgp-9a* (3-fold), *Hco-pgp-11* (4-fold) and *Hco-haf-*9 (2-fold) when comparing with the unselected ISE isolate (*p* < 0.05). Exposure of selected isolates to anthelmintics lead to no significant upregulation of the studied transporter genes. We also observed instances where there was strong intragroup variation regarding samples originating from parasites obtained from different individual hosts pointing that the interactions of the animal host with the tested anthelmintics may also play a role in the expression of the studied nematode genes.

## Introduction

Parasitism by gastrointestinal nematodes is one of the main causes of losses in small ruminant production systems (Shakya et al., 2017; Khan, et al., 2021). Control of these parasites can be carried out with the use of commercial anthelmintics, vaccination (Barbevax^®^) and through non-chemical control that concerns different management protocols (Arsenopoulos et al., 2021). However, control with anthelmintics such as benzimidazoles and macrocyclic lactones are still the main choice and the use of these drugs leads to the inevitable emergence of anthelmintic resistance (Geurden et al., 2014; Herrera-Manzanilla et al., 2017; Santos et al., 2017). *Haemonchus contortus* is an important trichostrongylid parasite due to the quick development of resistance to the main anthelmintic classes, significant economic losses in high prevalence regions worldwide, and aspects in its biology and physiology such as the high biotic potential and high pathogenicity (Gilleard, 2006, 2013). Anthelmintic resistance mechanisms are multifactorial and involve genetic alterations such as changes in the drug target, in its expression, and in drug distribution which prevent it from reaching its target. It is thought that resistance to ivermectin is polygenic (Lespine et al., 2012; Redman et al., 2012) while benzimidazole resistance is associated with polymorphisms in the beta-tubulin isotype 1 (Ghisi et al., 2007; Prichard, 2001; Silvestre and Cabaret, 2002) gene but it may also be associated with drug efflux transporters (Blackhall et al., 2008) thus being also polygenic.

ABC transporters are membrane proteins grouped into subclasses according to their structure and biological function. These proteins obtain energy from ATP hydrolysis to move a variety of compounds across biological membranes (Locher, 2016). The full structure of an ABC transporter consists of two nucleotide binding domains (NBD) and two transmembrane domains (TMD) while the structure of a half transporter is composed of only one NBD and one TMD and form dimers to become functional (Xiong et al., 2015). The P-glycoprotein transporters are of particular interest as they have been generally implicated in cases of drug resistance (Lespine et al., 2012; Ardelli and Prichard, 2013; Bygarski et al., 2014). In total, the *H. contortus* genome contains ten functional genes coding for P-glycoprotein transporters (*pgp*). Gene duplications in *C. elegans* (*pgp-3* and *pgp-4; pgp-12, pgp-13* and *pgp-14*) correspond to single genes in *H. contortus* (*pgp-3* and *pgp-13*). Two paralogue copies of *Cel-pgp-9* are present in the *H. contortus* genome (*pgp-9a* and *pgp-9b*) (Laing et al., 2013). Four *pgp* genes (*pgp-5, pgp-6, pgp-7 and pgp-8*) are absent in *H. contortus* and, in addition, the parasite genome encodes *Hco-pgp-16*, which is absent in *C. elegans* (Laing *et al.*, 2013). The HALF transporters, which are PGP-related, can also participate in anthelmintic resistance (Yan et al., 2012). In *C. elegans*, eight *haf* genes are found and only five of them are present in the *H. contortus* genome *(haf-2, haf-3, haf-4, haf-6 and haf-9*). Generally, many anthelmintics actively bind PGPs and, therefore, are amenable to removal through increased expression of this efflux pump (Kerboeuf et al., 2003; Godoy et al., 2015 b ; 2016; Kotze and Prichard, 2016; Gerhard et al., 2020). This study aimed to evaluate the expression levels of 6 PGP transporters and 1 half transporter in *H. contortus* isolates previously selected for oxfendazole (OXF) and ivermectin (IVM) resistance in comparison to the susceptible isolate ISE (pre-selection) as well as evaluating changes in gene expression of the same genes after exposure to these anthelmintics in comparison to non-exposed isolates.

## Material and Methods

### Animal welfare

The use of animals in this experiment was in accordance with internationally accepted guidelines for the experimental use of animals and was approved by the Embrapa Caprinos e Ovinos Local Ethics Committee Authorization Protocol number 010/2015. Animal maintenance and confinement conditions were as previously described (Santos et al., 2017). Before experimental infections, all lambs were treated with ivermectin (200 μg/kg) (Ivomec, Boehringer Ingelheim, Germany), oxfendazole (5 mg/kg) (Oxfaden, Biovet, Brazil), monepantel (2.5 mg/kg) (Zolvix, Novartis, Switzerland) and levamisole (7.5 mg/kg) (Ripercoll, Zoetis, U.S.A.) to become worm-free. Confirmation that the animals were free of gastrointestinal nematodes was done by eggs per gram (EPG) counts and fecal cultures.

### Isolates of *Haemonchus contortus*

The resistant *H. contortus* isolates used here were one selected for IVM resistance (IVM-ISE) and another selected for OXF resistance (OXF-ISE). The IVM-ISE isolate is resistant to both ivermectin and oxfendazole while the OXF-ISE isolate is resistant to oxfendazole only. These isolates were independently selected starting from the *Inbred Strain Edinburgh* (ISE) isolate as previously described (Santos et al., 2017). The *Haemonchus contortus* ISE isolate (Roos et al., 2004) was used for comparisons against the resistant isolates in the gene expression section of the study.

### Faecal egg count reduction test (FECRT)

The FECRT was performed to confirm the resistance status of the isolates used here. Two groups of worm-free six-month-old male lambs (n = 18 per group) were infected orally with 3,000 L3 larvae of IVM-ISE isolate for one group and with 3,000 L3 larvae of OXF-ISE isolate for the other. Two additional worm-free lambs were not infected in order to monitor any possible contamination from outside sources using weekly EPG counts and fecal cultures. Infection was monitored by EPG counts using the modified McMaster technique (Ueno and Gonçalves, 1998) and a multiplication factor of x25. Infected animals were further divided into four groups (n = 9 per group) according to anthelmintic treatments: groups A1 and A3 were treated with OXF (5 mg/kg) (Oxfaden, Biovet, Brazil) and groups A2 and A4 were treated with ivermectin (200 µg/kg) (Ivomec, Boehringer Ingelheim, Germany) (Table 1). Groups were placed in individual stalls with raised slatted floors. Feces for EPG counts and efficacy calculations were collected before and after treatment on days eight for groups treated with OXF and fourteen for groups treated with IVM (Coles et al., 2006). 

**Table 1 -:** Experimental design for the fecal egg count reduction test showing the *H. contortus* isolates used and anthelmintic treatments with ivermectin and oxfendazole.

Treatment groups for FECRT (n = 9 animals per group)	
Animals infected with the IVM-ISE isolate	Animals infected with the OXF-ISE isolate
A1: OXF Treatment (5 mg/kg)	A3: OXF Treatment (5 mg/kg)
A2: IVM Treatment (200 µg/kg)	A4: IVM Treatment (200 µg/kg)

### Experimental design for gene expression study

Nine male lambs infected with IVM-ISE (3,000 L3 larvae per animal) were divided into three groups (n = 3) and six male lambs infected with OXF-ISE (3,000 L3 larvae per animal) were divided into two groups (n = 3). These groups were treated with IVM (Ivomec, Boehringer Ingelheim, Germany) or OXF (Oxfaden, Biovet, Brazil) as shown in Table 2, with controls without treatment for each isolate. Another group with 3 male lambs was infected with the ISE isolate (3,000 L3 larvae per animal). Two animals from each group were euthanized between 12 to 14 hours after dosing with anthelmintics. This timing was selected in order to expose the parasites to concentrations close to the peak plasma levels (Alvinerie et al., 2008; Viviani et al., 2019). Two additional worm-free lambs were not infected in order to monitor any possible contamination from outside sources using weekly EPG counts and fecal cultures. All parasites were collected from the abomasum of each animal, stored in RNAlater (Invitrogen, Carlsbad, CA, USA) and counted. The parasites were separated in males and females using optic microscopy and stored in RNAlater (Invitrogen, Carlsbad, CA, USA) at -80 °C until total RNA extraction. Thus, this experiment analyzes two biological events: selection for resistance comparing the untreated groups with the ISE group and drug exposure comparing treated groups with untreated ones (Table 2). It must be noted that the ISE infected group, euthanasia and parasite collection were done three months before the remaining groups due to infrastructure limitations. However, the entire study occurred during the same season of the year (dry season) in order to minimize environmental effects that may affect gene expression in this experiment.

**Table 2:** *Haemonchus contortus* isolates used in the experimental groups for gene expression analysis and the anthelmintic treatments used.

Susceptible isolate.	Selected for anthelmintic resistance.	Resistant isolates exposed to anthelmintics.
ISE (Inbred Strain Edinburgh).	B1: IVM-ISE isolate no treatment.	B2: IVM-ISE isolate exposed to IVM (200 µg/host kg).
		B3: IVM-ISE isolate exposed to OXF (5 mg/host kg).
	C1: OXF-ISE isolate no treatment.	C2: OXF-ISE isolate exposed to OXF (5 mg/host kg).

### Nucleic acids extraction

Genomic DNA was extracted from 20,000 ISE L3 larvae as previously described (Santos et al., 2014). Total RNA was extracted from three pools of approximately 20 males of *H. contortus* from each euthanized animal from each experimental group (2 lambs/group). The RNA extraction was performed with the Trizol reagent (Invitrogen, Carlsbad, CA, USA) according to the manufacturer’s instructions with the following modifications in material disruption: adult males were suspended in Trizol and physically disrupted by shaking with 1 mm zirconia/silica beads in a Mini-BeadBeater-16 (Biospec Products, Bartlesville, OK, USA) for four 20 second runs. The integrity of the RNA samples was confirmed by visualization of 28S and 18S ribosomal bands under an UV light source after electrophoresis in 1.5% agarose gel stained with ethidium bromide. Samples concentration and purity were determined by UV spectrophotometry at 260 and 280 nm (i-Quant, Loccus, Brazil) and stored at -80 °C. 

### Primers and establishment of RT-qPCR

The NCBI nucleotide database and the literature were searched in order to identify all gene sequences of interest, including the available *H. contortus* six *pgps* and one *haf* transporter which may be involved in anthelmintic resistance (Yan et al., 2012). GenBank accession numbers for all studied genes were obtained, and the BLASTN tool (Altschul et al., 1990) available at the *H. contortus* genome database (WormBase ParaSite BioProject PRJEB506) was used to find the genomic sequences for the studied genes. At least one primer from each pair was designed to anneal across the border of adjacent exons to prevent amplification of DNA that may have been extracted along with RNA (Primer3Plus) (Untergasser et al., 2007). Glyceraldehyde-3-phosphate dehydrogenase (*Hco-gapdh*), actin 1 (*Hco-act-1*), ribosomal protein L9 (*Hco-prl9*) and tyrosine-3-monoxygenase (*Hco-ftt-2*) were selected as candidate reference genes and primers for these were designed in the same manner as described above (Lecová et al., 2015; Toscano et al., 2018) with the exception of the actin gene where the primer sequences were obtained from the literature (Maté et al., 2018).

Efficiency curves were assembled to test the designed primers. Complementary DNA samples from the ISE isolated were synthesized with the SuperScript ™ IV VILO ™ Master Mix two-step kit according to the manufacturer’s instructions (Invitrogen, Carlsbad, CA, USA) on a Mastercycler ep Gradient S Realplex^4^ (Eppendorf, Germany) thermocycler. Datapoints consisted of reactions containing serial dilutions based in the amount of total RNA used in cDNA synthesis (0.8-500 ng/rxn in five-fold increments). The percentage amplification efficiency of each target was determined according to equation E = 10^(-1/S)^-1, where S was the slope of the standard curve generated from quantification cycle (Cq) values of each reaction for a given primer pair. In addition, primers were tested with *H. contortus* gDNA to confirm its specificity, and reaction products were visualized under an UV light source, after electrophoresis, on ethidium bromide stained 1.5% agarose gels. Polymerase chain reactions using gDNA were assembled as follows: 12.5 μL 2x Fast Start Universal SYBR Green Master Mix (Roche, West Sussex, UK), 100 nM of each primer (forward and reverse), 25 ng of total DNA and water for final volume of 25 μL. Amplification conditions were: 95 °C for 10 min and 35 cycles at 95 °C for 10 s, 58 °C for 30 s and extension at 72 °C for 30 s. Melting curve analysis was applied to detect primer dimers.

### Gene expression analysis

All RT-qPCR tests were performed in triplicate using the GoTaq® 1-Step RT-qPCR System reverse transcriptase kit (Promega, Madison, Wisconsin, USA) following the manufacturer’s protocol. Reactions contained 2X GoTaq^®^ qPCR Master Mix, 50X GoScript™ RT Mix for 1-Step RT-qPCR, 100 nM of each primer (forward and reverse), 100 ng of total RNA and water for a final volume of 25 μL. Negative controls used water instead of RNA. Amplification conditions were: 37 °C for 15 min, 95 °C for 10 min and 35 cycles at 95 °C for 10 s, 58 °C for 30 s and extension at 72 °C for 30 s. Melting curve analysis was applied to detect primer dimers. Amplified product lengths were confirmed on ethidium bromide stained 1.5% agarose gels after electrophoresis and visualized on an UV light source.

### Data analysis

Anthelmintic efficiency was calculated based on EPG data before and after treatment using a Bayesian hierarchical model in the egg counts software available at http://shiny.math.uzh.ch/user/furrer/shinyas/shiny-eggCounts/ (Torgerson et al., 2014). 

The Cq values for RT-qPCR reactions were determined by the software Realplex 2.2 (Eppendorf, Hamburg, Germany). The stability of the four candidate reference genes was tested using pair-wise correlation analysis by the Bestkeeper software version 1 (Pfaffl et al., 2004). Normalized relative expression was calculated per animal and then per experimental group as previously described (Taylor et al., 2019).

Data normality was verified using the Shapiro-Wilk test (Shapiro and Wilk, 1965). Relative gene expression statistical analysis consisted of a one-way ANOVA test with Bonferroni’s correction for multiple comparisons for the selection experiment comparing ISE with B1 and C1 and the exposure experiment comparing B1 with B2 and B3. Student’s *t*-test was used for the exposure experiment comparing C1 and C2 (Graphpad Prism Software 6.05, La Jolla, CA, USA) (Table 2). A value of *p* < 0.05 was considered for statistical significance.

## Results

### Faecal egg count reduction test (FECRT)

The results of the FECRT are shown in Table 3. Group A1 showed an increase in egg counts. In contrast, the other groups showed a decrease in egg counts, especially the group A4, which showed a high decrease (97%).

**Table 3 -:** Fecal egg count reduction test results, EPG count means before and after treatment, efficacy and 95% confidence interval in parentheses.

Groups	EPG Means			% Efficacy (95% CI) FECRT
	Day 0	Day 8	Day 14	
A1^¹^	2525	4758.3	-	0 (-8 : -228)
A2^¹^	2338.9	-	1877.7	21 (-52 : -59)
A3 ^¹^	7603.1	5718.8	-	25 (-51 : 63)
A4 ^¹^	7482.1	-	200	97 (89 : -99)

^¹^ A1: IVM-ISE infected lambs treated with OXF (5 mg/kg); A2: IVM-ISE infected lambs treated with IVM (200 µg/kg); A3: OXF-ISE infected lambs treated with OXF (5 mg/kg); A4: OXF-ISE infected lambs treated with IVM (200 µg/kg).

### RT-qPCR evaluation

Accession numbers for the *Haemonchus contortus* complete genes used in primer design are shown in Table S1. All used primer pairs showed amplification efficiencies above 90% (Table 4) and PCR reactions using gDNA showed no amplification, confirming their specificity for RNA. Only one band was visualized after agarose gel electrophoresis of all reverse transcribed amplified products, demonstrating the specificity of the assays (Figures S1, S2, S3, S4 and S5). 

**Table 4 -:** Primer sequences used in the RT-qPCR assays, predicted product lengths and validation results. F: foward and R: reverse.

Gene	Primer sequences (5’ to 3’)	Product size (bp^¹^)	Reaction efficiency	Linear dynamic range	r^2^
*Hco-pgp-2*	F: AGGATGGTGTCACGAAGAAAAT	172	100%	26.91-34.89	0.965
	R: GCCATCACAGTGCTTTTTCC				
*Hco-pgp-3*	F: TCAAAGTCGTGCAGATCGAG	107	99%	23.05-32.50	0.993
	R: AGTTGTCACGAGCACTTTCA				
*Hco-pgp-9a*	F: ACGTGAAGTGAACCCAACTC	197	91%	24.13-32.16	0.989
	R: TGTTGTAACCATCTGGCAGG				
*Hco-pgp-10*	F: CAGAAAGATTATGCGCCACG	199	104%	26.55-31.94	0.954
	R: GAGTGCCATCTTCCAGTTGA				
*Hco-pgp-11*	F: GTTTTGGTGGATGGTCAGGT	166	95%	22.63-32.78	0.996
	R: TCGAGACACGCTTGAACATC				
*Hco-pgp-16*	F: TTGAATCCTTGAACACCGCG	164	91%	23.32-30.92	0.994
	R: TCCGCGTAACTTAGTTTGCA				
*Hco-haf-9*	F: CAGGTCTGTCACGGAAAACA	170	94%	20.78-30.67	0.991
	R: GTTGCTTTTGTCCACCTGAC				
*Hco-gapdh*	F: GAGCACTCACAGGATCAAGG	163	95%	15.40-25.32	0.993
	R: AGCAGAGATGACGACCTTCT				
*Hco-act*	F: GCTCCCAGCACGATGAAAA	100	96%	18.20-28.06	0.985
	R: CACCAATCCAGACAGAGTATTTGC				
*Hco-prl9*	F: TCAAGGGTGTCACTAAGGGT	115	91%	16.20-26.22	0.985
	R: CCGAGGAAGTTACGAATCTCA				
*Hco-ftt-2*	F: CGATTGAGCAGAAGACGGAA	113	98%	14.24-23.99	0.995
	R: GAGCAGGTTCAAAACGTCCT				

^¹^ base pairs

### Gene expression

BestKeeper analysis results determined *Hco-gapdh, Hco-prl9* and *Hco-ftt-2* as the most stable genes with low coefficients of variation, unlike *Hco-act-1* which presented a high coefficient of variation (CV = 5.59%). Therefore, *Hco-gapdh, Hco-prl9* and *Hco-ftt-2* were considered as the reference genes for relative gene expression calculations in this study. All used primers were evaluated by melting curve analysis resulting in single defined peaks at the expected melting temperature of the PCR products (data not shown).

**Figure 1 -:**
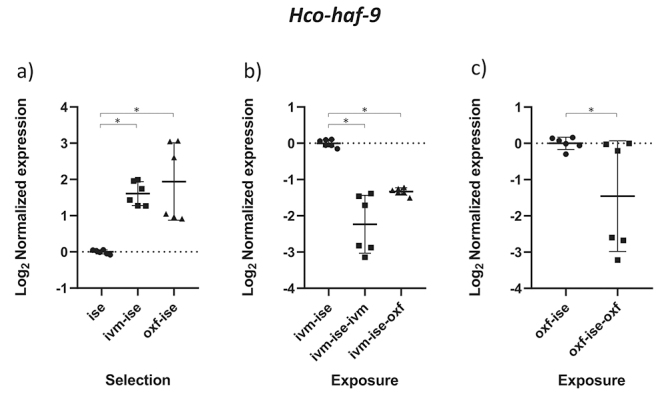
Scatter plot showing relative expression of the gene *Hco-haf-9* of *Haemonchus contortus* adult males. Relative gene expression values are shown on the Y axis. The X axis contains the different groups studied: a) the Inbred Strain Edinburgh (ISE) isolate is the unselected control, isolate ISE selected for IVM resistance (IVM-ISE), isolate ISE selected for OXF resistance (OXF-ISE); b) IVM-ISE isolate treated with IVM (200 µg/host kg) (IVM-ISE-IVM), IVM-ISE isolate treated with OXF (5 mg/host kg) (IVM-ISE-OXF); c) OXF-ISE isolate treated with OXF (5 mg/host kg) (OXF-ISE-OXF). All treated groups were sampled at 12-14 hours after the hosts received the anthelmintic. Relative gene expression statistical analysis consisted of a one-way ANOVA test with Bonferroni’s correction for multiple comparisons for the selection experiment comparing ISE, IVM-ISE and OXF-ISE and the exposure experiment comparing IVM-ISE, IVM-ISE-IVM and IVM-ISE-OXF. Student’s *t*-test was used for the exposure experiment comparing OXF-ISE and OXF-ISE-OXF. Asterisks represent significant differential gene expression levels between compared groups (*p* < 0.05).

**Figure 2 -:**
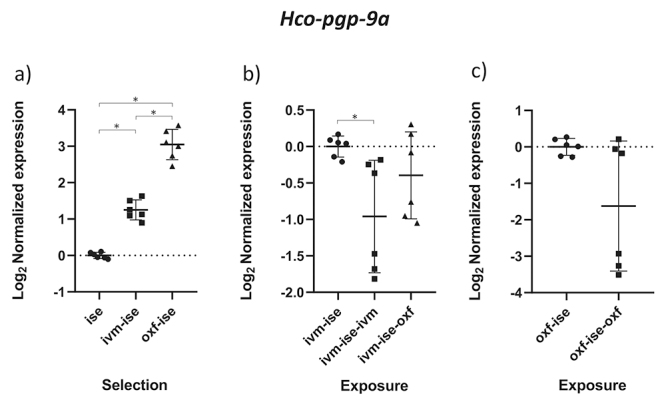
Scatter plot showing relative expression of the gene *Hco-pgp-9a* of *Haemonchus contortus* adult males. Relative gene expression values are shown on the Y axis. The X axis contains the different groups studied: a) the Inbred Strain Edinburgh (ISE) isolate is the unselected control, isolate ISE selected for IVM resistance (IVM-ISE), isolate ISE selected for OXF resistance (OXF-ISE); b) IVM-ISE isolate treated with IVM (200 µg/host kg) (IVM-ISE-IVM), IVM-ISE isolate treated with OXF (5 mg/host kg) (IVM-ISE-OXF); c) OXF-ISE isolate treated with OXF (5 mg/host kg) (OXF-ISE-OXF). All treated groups were sampled at 12-14 hours after the hosts received the anthelmintic. Relative gene expression statistical analysis consisted of a one-way ANOVA test with Bonferroni’s correction for multiple comparisons for the selection experiment comparing ISE, IVM-ISE and OXF-ISE and the exposure experiment comparing IVM-ISE, IVM-ISE-IVM and IVM-ISE-OXF. Student’s *t*-test was used for the exposure experiment comparing OXF-ISE and OXF-ISE-OXF. Asterisks represent significant differential gene expression levels between compared groups (*p* < 0.05).

**Figure 3 -:**
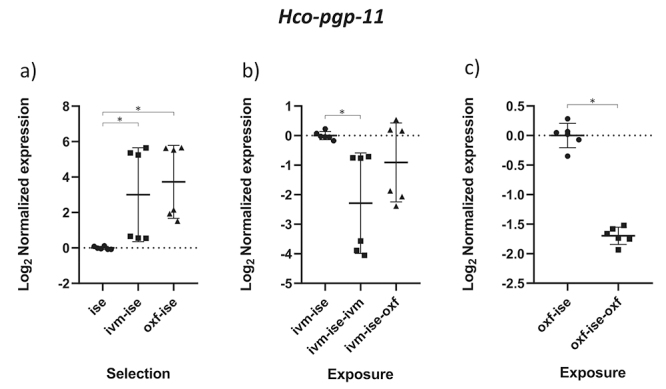
Scatter plot showing relative expression of the gene *Hco-pgp-11* of *Haemonchus contortus* adult males. Relative gene expression values are shown on the Y axis. The X axis contains the different groups studied: a) the Inbred Strain Edinburgh (ISE) isolate is the unselected control, isolate ISE selected for IVM resistance (IVM-ISE), isolate ISE selected for OXF resistance (OXF-ISE); b) IVM-ISE isolate treated with IVM (200 µg/host kg) (IVM-ISE-IVM), IVM-ISE isolate treated with OXF (5 mg/host kg) (IVM-ISE-OXF); c) OXF-ISE isolate treated with OXF (5 mg/host kg) (OXF-ISE-OXF). All treated groups were sampled at 12-14 hours after the hosts received the anthelmintic. Relative gene expression statistical analysis consisted of a one-way ANOVA test with Bonferroni’s correction for multiple comparisons for the selection experiment comparing ISE, IVM-ISE and OXF-ISE and the exposure experiment comparing IVM-ISE, IVM-ISE-IVM and IVM-ISE-OXF. Student’s *t*-test was used for the exposure experiment comparing OXF-ISE and OXF-ISE-OXF. Asterisks represent significant differential gene expression levels between compared groups (*p* < 0.05).

**Figure 4 -:**
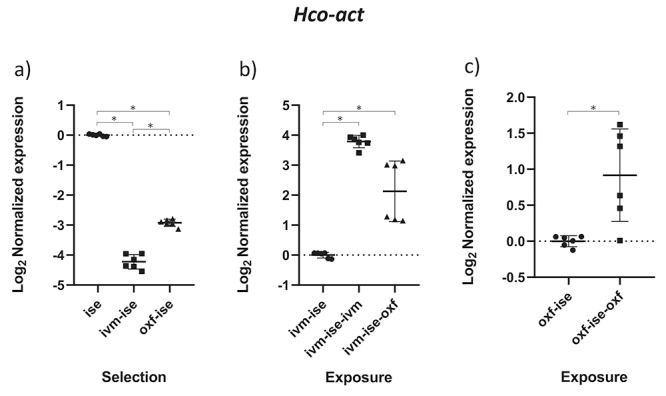
Scatter plot showing relative expression of the gene *Hco-act*-1 of *Haemonchus contortus* adult males. Relative gene expression values are shown on the Y axis. The X axis contains the different groups studied: a) the Inbred Strain Edinburgh (ISE) isolate is the unselected control, isolate ISE selected for IVM resistance (IVM-ISE), isolate ISE selected for OXF resistance (OXF-ISE); b) IVM-ISE isolate treated with IVM (200 µg/host kg) (IVM-ISE-IVM), IVM-ISE isolate treated with OXF (5 mg/host kg) (IVM-ISE-OXF); c) OXF-ISE isolate treated with OXF (5 mg/host kg) (OXF-ISE-OXF). All treated groups were sampled at 12-14 hours after the hosts received the anthelmintic. Relative gene expression statistical analysis consisted of a one-way ANOVA test with Bonferroni’s correction for multiple comparisons for the selection experiment comparing ISE, IVM-ISE and OXF-ISE and the exposure experiment comparing IVM-ISE, IVM-ISE-IVM and IVM-ISE-OXF. Student’s *t*-test was used for the exposure experiment comparing OXF-ISE and OXF-ISE-OXF. Asterisks represent significant differential gene expression levels between compared groups (*p* < 0.05).

**Figure 5 -:**
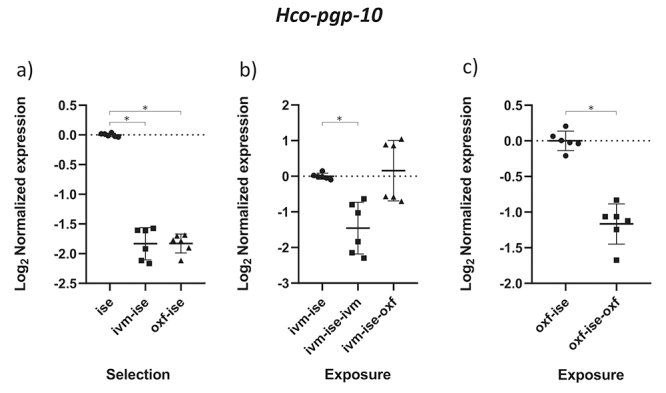
Scatter plot showing relative expression of the gene *Hco-pgp-10* of *Haemonchus contortus* adult males. Relative gene expression values are shown on the Y axis. The X axis contains the different groups studied: a) the Inbred Strain Edinburgh (ISE) isolate is the unselected control, isolate ISE selected for IVM resistance (IVM-ISE), isolate ISE selected for OXF resistance (OXF-ISE); b) IVM-ISE isolate treated with IVM (200 µg/host kg) (IVM-ISE-IVM), IVM-ISE isolate treated with OXF (5 mg/host kg) (IVM-ISE-OXF); c) OXF-ISE isolate treated with OXF (5 mg/host kg) (OXF-ISE-OXF). All treated groups were sampled at 12-14 hours after the hosts received the anthelmintic. Relative gene expression statistical analysis consisted of a one-way ANOVA test with Bonferroni’s correction for multiple comparisons for the selection experiment comparing ISE, IVM-ISE and OXF-ISE and the exposure experiment comparing IVM-ISE, IVM-ISE-IVM and IVM-ISE-OXF. Student’s *t*-test was used for the exposure experiment comparing OXF-ISE and OXF-ISE-OXF. Asterisks represent significant differential gene expression levels between compared groups (*p* < 0.05).

**Figure 6 -:**
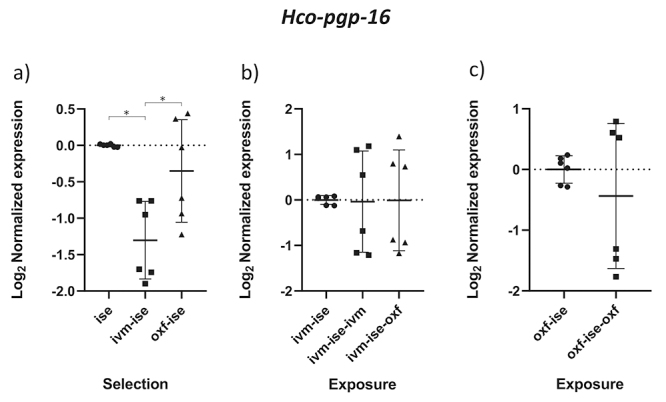
Scatter plot showing relative expression of the gene *Hco-pgp-16* of *Haemonchus contortus* adult males. Relative gene expression values are shown on the Y axis. The X axis contains the different groups studied: a) the Inbred Strain Edinburgh (ISE) isolate is the unselected control, isolate ISE selected for IVM resistance (IVM-ISE), isolate ISE selected for OXF resistance (OXF-ISE); b) IVM-ISE isolate treated with IVM (200 µg/host kg) (IVM-ISE-IVM), IVM-ISE isolate treated with OXF (5 mg/host kg) (IVM-ISE-OXF); c) OXF-ISE isolate treated with OXF (5 mg/host kg) (OXF-ISE-OXF). All treated groups were sampled at 12-14 hours after the hosts received the anthelmintic. Relative gene expression statistical analysis consisted of a one-way ANOVA test with Bonferroni’s correction for multiple comparisons for the selection experiment comparing ISE, IVM-ISE and OXF-ISE and the exposure experiment comparing IVM-ISE, IVM-ISE-IVM and IVM-ISE-OXF. Student’s *t*-test was used for the exposure experiment comparing OXF-ISE and OXF-ISE-OXF. Asterisks represent significant differential gene expression levels between compared groups (*p* < 0.05).

The results obtained by the normality test indicated that the data followed a normal distribution (p < 0.05). The comparison between groups B1 (IVM-ISE) and C1 (OXF-ISE) with the ISE isolate addresses the selection process for anthelmintic resistance (Table 2). Relative profiles of gene expression in adult male parasites in groups B1 and C1 showed significant changes compared to the ISE control (*p* < 0.05). *Hco-haf-9*, *Hco-pgp-9a* and *Hco-pgp-11* were upregulated in both selection groups (Figures 1 a , 2a and 3a). *Hco-haf-9* about 1.5-fold in B1 and 2-fold in C1, *Hco-pgp-9*a about 1.5-fold in B1 and 3-fold in C1 and *Hco-pgp-11* about 3-fold in B1 and ~4-fold in C1. On the other hand, *Hco-act-1* and *Hco-pgp-10* were downregulated more than 2-fold in both selection groups (Figures 4 a  and 5a). *Hco-pgp-16* was downregulated ~1.5-fold in the ivermectin selection group (Figure 6 a ). Direct comparison between the selected groups B1 and C1 showed statistically significant differences in the expression of *Hco-act-1*, *Hco-pgp-9a* and *Hco-pgp-16* (p < 0.05 ) (Figures 2 a , 4a and 6a). 

**Figure 7 -:**
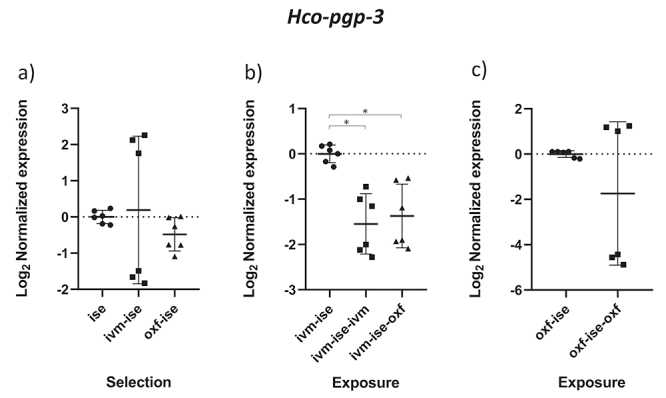
Scatter plot showing relative expression of the gene *Hco-pgp-3* of *Haemonchus contortus* adult males. Relative gene expression values are shown on the Y axis. The X axis contains the different groups studied: a) the Inbred Strain Edinburgh (ISE) isolate is the unselected control, isolate ISE selected for IVM resistance (IVM-ISE), isolate ISE selected for OXF resistance (OXF-ISE); b) IVM-ISE isolate treated with IVM (200 µg/host kg) (IVM-ISE-IVM), IVM-ISE isolate treated with OXF (5 mg/host kg) (IVM-ISE-OXF); c) OXF-ISE isolate treated with OXF (5 mg/host kg) (OXF-ISE-OXF). All treated groups were sampled at 12-14 hours after the hosts received the anthelmintic. Relative gene expression statistical analysis consisted of a one-way ANOVA test with Bonferroni’s correction for multiple comparisons for the selection experiment comparing ISE, IVM-ISE and OXF-ISE and the exposure experiment comparing IVM-ISE, IVM-ISE-IVM and IVM-ISE-OXF. Student’s *t*-test was used for the exposure experiment comparing OXF-ISE and OXF-ISE-OXF. Asterisks represent significant differential gene expression levels between compared groups (*p* < 0.05).

**Figure 8 -:**
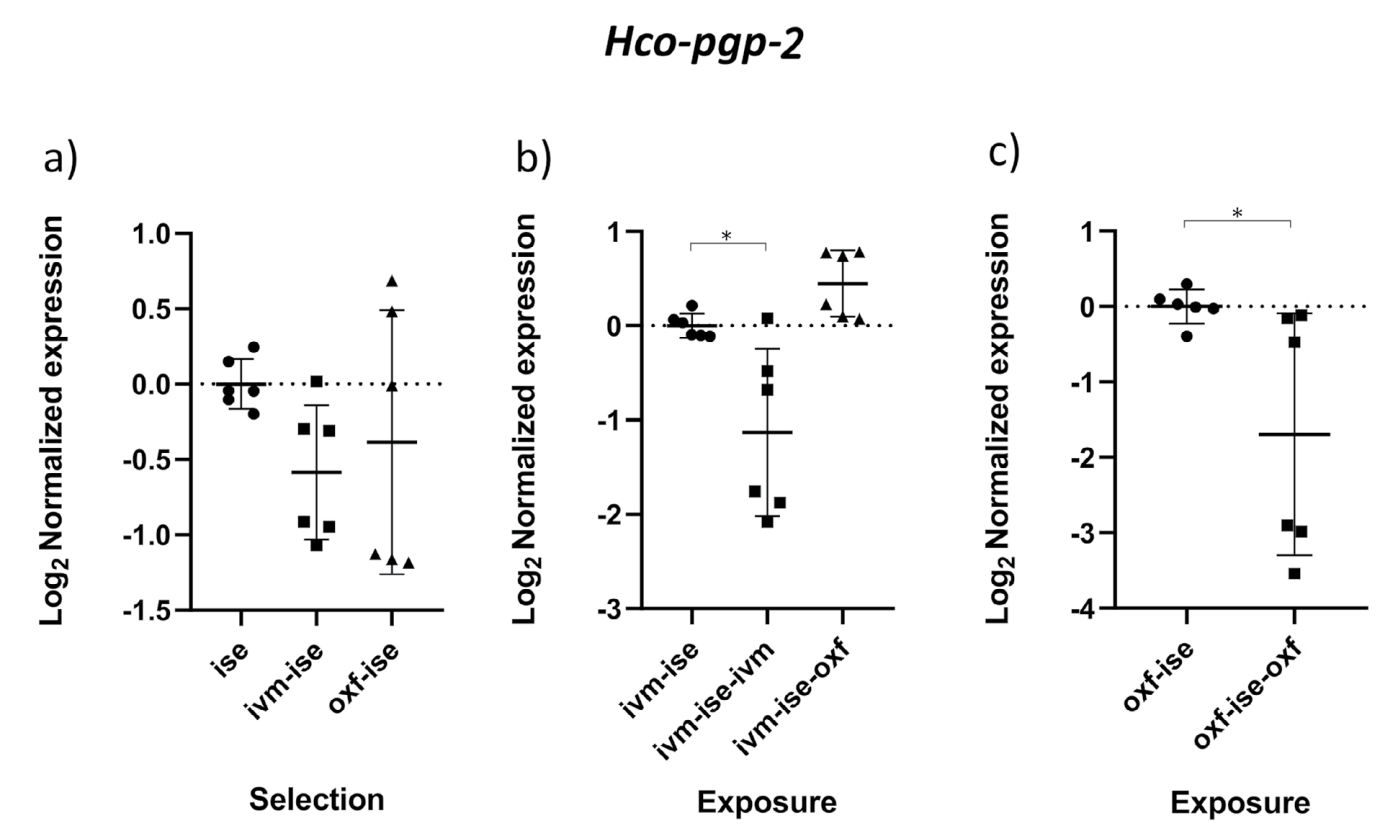
Scatter plot showing relative expression of the gene *Hco-pgp-2* of *Haemonchus contortus* adult males. Relative gene expression values are shown on the Y axis. The X axis contains the different groups studied: a) the Inbred Strain Edinburgh (ISE) isolate is the unselected control, isolate ISE selected for IVM resistance (IVM-ISE), isolate ISE selected for OXF resistance (OXF-ISE); b) IVM-ISE isolate treated with IVM (200 µg/host kg) (IVM-ISE-IVM), IVM-ISE isolate treated with OXF (5 mg/host kg) (IVM-ISE-OXF); c) OXF-ISE isolate treated with OXF (5 mg/host kg) (OXF-ISE-OXF). All treated groups were sampled at 12-14 hours after the hosts received the anthelmintic. Relative gene expression statistical analysis consisted of a one-way ANOVA test with Bonferroni’s correction for multiple comparisons for the selection experiment comparing ISE, IVM-ISE and OXF-ISE and the exposure experiment comparing IVM-ISE, IVM-ISE-IVM and IVM-ISE-OXF. Student’s *t*-test was used for the exposure experiment comparing OXF-ISE and OXF-ISE-OXF. Asterisks represent significant differential gene expression levels between compared groups (*p* < 0.05).

Comparisons between groups B2 (ivermectin treated IVM-ISE) and B3 (oxfendazole treated IVM-ISE) with B1 (untreated IVM-ISE) and C2 (oxfendazole treated OXF-ISE) with C1 (untreated OXF-ISE) reflect exposure to anthelmintics. In general, gene expression levels did not show significant upregulation due to exposure to anthelmintics with the exception of *Hco-act-1*. This gene showed significant upregulation upon exposure of B1 to anthelmintics, with a ~4-fold increase in expression in the B2 group and a 2-fold increase in expression in the B3 group (Figure 4 b  and 4c). *Hco-haf-9* also showed significant changes in expression in all groups exposed to anthelmintics, ranging from a 1.5 drop in expression in group C2 to a 2.3 drop in expression in group B2 (Figures 1 b  and 1c). *Hco-pgp-3* showed changes in B2 and B3 with a drop in expression of ~1.5 fold (Figures 7 b  and 7c). *Hco-pgp-2*, *Hco-pgp-9a*, *Hco-pgp-10* and *Hco-pgp-11* showed a significant drop in expression in B2 and C2 (Figures 2, 3, 5 and 8). We also observed a strong variation in results when analyzing gene expression in *H. contortus* males obtained from different hosts from the same groups which can be seen in the Figures as very wide standard deviation intervals. 

## Discussion

Fecal egg count reduction results confirmed *in vivo* the resistance status of the isolates resulting from the selection process carried out previously (Santos et al., 2017). The selection of *H. contortus* ISE for IVM resistance led to the development of OXF and IVM resistance. However, the selection of *H. contortus* ISE for OXF led to OXF resistance only. It has been shown that ivermectin can bind tubulin and affect cytoskeleton dynamics (Ashraf et al., 2015). In addition, the SNP F200Y has been detected in macrociclic lactone resistant individuals that have never been exposed to benzimidazoles (Mottier and Prichard, 2008). Benzimidazoles and macrocyclic lactones are commonly used in the field in Brazil and alternations between these two classes can contribute to the maintenance of high levels of BZ resistance seen in populations of *H. contortus* (Santos *et al.*, 2017). 

In our study, the *Hco-act-1* gene was not suitable as a control gene due to a high coefficient of variation among different groups. In this context, another study of p-glycoprotein gene expression in *H. contortus* also showed actin as a least stable candidate reference gene (Giglioti et al., 2022). In our case, this is not surprising as the anthelmintics studied here are known to interact with the microtubular component of the cytoskeleton. It should be expected that disturbances in the microtubules may impact microfilament dynamics as well since it has been shown that microtubules interact with microfilaments with impacts in three-dimensional cell structure among other cellular activities (Griffith and Pollard, 1982; Dugina et al., 2016). While these studies describe interactions at the protein level, it may be possible that interactions between the tested anthelmintics and tubulin microtubules will disrupt the microtubular cytoskeleton (Lacey, 1988; Kohler, 2001) and lead to gene expression changes in other genes coding for cytoskeletal proteins such as actin. In addition, gene expression changes in actin have been reported in different life stages of *Teladorsagia circumcincta* albeit not between ivermectin resistant and susceptible isolates (Dicker et al., 2011). We observed significant changes in actin gene expression both due to selection (downregulation) and anthelmintic exposure (upregulation) which suggest that cytoskeleton protein coding genes may not be the best controls when analyzing gene expression changes induced by compounds that interact with cytoskeleton components.

The interaction of PGPs with ivermectin have been previously demonstrated in *Haemonchus contortus* (Prichard and Roulet, 2007; Godoy et al., 2015 b ) in addition to their upregulation in ivermectin resistant field strains (Xu et al., 1998). The role of PGPs in benzimidazole resistance has also been considered in multidrug-resistant cells as interactions were detected between BZs and PGPs (Nare et al., 1994). Furthermore, the linker domain present in many ABC transporters was also shown to bind α- and β-tubulin in drug-resistant tumor cell lines (Georges, 2007). BZs are known to target β-tubulin in helminths and resistance to this class of drugs is associated with aminoacid changes in this protein (Robinson et al., 2004; Kotze and Prichard, 2016). In *H. contortus* specifically, polymorphism selection of P-glycoprotein locus in a cambendazole resistant strain and another thiabendazole resistant strain has been detected (Blackhall et al., 2008). It was also shown, using the egg hatch assay, that the PGP inhibitor verapamil can increase BZ toxicity in BZ resistant *H. contortus* isolates (Beugnet et al., 1997) suggesting that the involvement of these proteins in benzimidazole resistance is possible. On the other hand, another study using pig kidney epithelial cell lines overexpressing a murine PGP transporter, while detecting some interaction with benzimidazoles, failed to detect specific interactions between PGP and oxfendazole (Dupuy et al., 2010). One last *in vitro* study using transduced MDCK II cell lines detected interactions between oxfendazole and breast cancer resistant human protein BCRP, another type of ABC transporter (Merino et al., 2005). Thus, the role of PGP in BZ resistance cannot be totally discarded.

*Hco-pgp-2* was the first PGP associated with IVM resistance in *H. contortus* eggs using isolates selected (MKIR) and unselected (MKIS) for resistance (Xu et al., 1998). *Hco-pgp-2* is expressed in the pharynx and adjacent nervous tissue, suggesting an important role in protecting nematode tissues against the effects of anthelmintics (Godoy et al., 2015 a ). Recently, it has been shown in an isolate resistant to anthelmintics, including ivermectin, that *Hco-pgp-2* expression is slightly higher in eggs and L1 larvae than other life stages of this parasite in comparison with the susceptible isolate (Giglioti et al., 2022). Adult females selected for IVM resistance in Argentina showed upregulation of *Hco-pgp-2* when compared to a local susceptible isolate and that gene expression was upregulated in resistant females after IVM exposure (Lloberas et al., 2013). On the other hand, ivermectin exposure of resistant adult *H. contortus* females in Argentina showed an increase in *Hco-pgp-2* expression but the results were not significant (Maté et al., 2018). Both mentioned studies used whole adult females including eggs, which may have influenced their results, in addition to the high genetic variability found in *H. contortus* populations (Gilleard and Redman, 2016). In the case of L3 larvae, there were no differences in expression between ISE-derived resistant and ISE susceptible isolates (Williamson and Wolstenholme, 2012). *Hco-pgp-2* expression in adult males presented here showed high intragroup variation especially from samples from different host animals. Although we observed a significant drop in expression after exposure to IVM and OXF in their own selection groups, we did not find significant changes caused by IVM and OXF selection (Figure 8). Likewise, *Hco-pgp-3* was downregulated by exposure to anthelmintics but only in the IVM-selected isolate, showing wide variation between samples from different host animals (Figure 7). *Hco-pgp-*3 was downregulated at 12 and 24 hours in resistant adult *H. contortus* females from Argentina after exposure to IVM (Maté *et al.*, 2018) but these results were not significant. In *C. elegans* this protein localizes in the gut and appears to be involved in resistance against heavy metals, chloroquine, colchicine and pyocyanin-dependent killing by *Pseudomonas aeruginosa* (Lincke et al., 1993; Broeks et al., 1995; Broeks *et al.*, 1996; Lindblom et al., 2001). Indeed, increased levels of *Hco-pgp-3* have been observed in four different resistant isolates in first-stage larvae (Sarai et al., 2013; Giglioti *et al.*, 2022). Clearly, this protein has more important roles in *H. contortus* free-living stages where it may come in contact with adverse compounds and toxin producing microorganisms.

Anthelmintic resistance selection led to *Hco-pgp-9a* upregulation with stronger effects for OXF resistance selection (Figure 2 a ). In terms of exposure to anthelmintics, expression levels did not show statistically significant differences except in the IVM-exposed IVM-selected isolate, where it showed a drop in expression (Figure 2 b ). Similarly, it has been shown that *Hco-pgp-9a* was downregulated in adult females from Argentina after IVM dosing (Maté et al., 2018) but in another study drug exposure did not significantly alter gene expression (Maté *et al.*, 2022). In *Teladorsagia circumcincta* this gene was shown to be upregulated in a multidrug resistant isolate (Dicker et al., 2011) which matches our observations when comparing selected and non-selected isolates (Figure 2 a ).

Little is known about *pgp-10* gene expression in *H. contortus* and what is known addresses its expression changes along the parasite life cycle. It was shown that this gene is overexpressed in Weybridge isolate L3 larvae in relation to its expression in eggs (Issouf et al., 2014). Simlarly, it is also shown to be upregulated in dauer stages in *C. elegans* (https://wormbase.org/species/c_elegans/gene/WBGene00004004#0c1-9gb6-10). In addition, an increase in the expression of this gene was observed in first-stage larvae in IVM-resistant isolates (Giglioti et al., 2022). In this work, we observed a significant downregulation of this gene in the selection groups versus the unselected ISE isolate. Exposure to anthelmintics caused a drop in gene expression (Figure 5). This indicates that the contribution of this gene to OXF and IVM resistance in adults could be rather small and that its roles may be more important in life stages where metabolic activity is rather decreased. 

Like *Hco-pgp-9a*, *Hco-pgp-11* showed positive regulation in terms of selection, but we observed a variation in expression results between samples originating from nematodes from different animals, except for the ISE isolate (Figure 3 a ). In terms of exposure to anthelmintics, negative regulation was observed in the same way as in *Hco-pgp-2* (Figures 3 b  and 3c). *Parascaris equorum* exposed *in vitro* to IVM showed no changes in the expression of this gene but SNPs potentially associated with IVM resistance were identified (Janssen et al., 2013). Thus, we cannot completely discard *pgps* showing little or no change in gene expression both for selection or exposure such as *Hco-pgp-2*, and *Hco-pgp-3* from participating in anthelmintic resistance as no sequencing was done in this study in order to detect resistance associated polymorphisms in the analyzed genes. 

*Hco-pgp-16* was ~1.5-fold downregulated as a result of IVM resistance selection, and we observed strong intragroup variation in expression for this gene when looking at samples from different animals. This gene was shown to be overexpressed in Weybridge isolate adult males in response to eosinophil granule proteins and it was also shown to be involved with macrocyclic lactone transport in the PF 23 susceptible strain (Issouf et al., 2014; Godoy et al., 2015 b ). We believe that further characterization is necessary in order to clarify the real association of this gene and anthelmintic resistance.

*Hco-haf-9* transporter was upregulated by anthelmintic selection while exposure led to downregulation (Figure 1). In contrast, the role of different ABC transporters, including *haf-9*, was evaluated in a *C. elegans* strain selected for IVM resistance. While several ABC transporters were overexpressed in the IVM resistant strain, it was not the case for *haf-9*. Downregulation of *haf-9* reduced egg production, but increased motility, at the highest IVM concentrations tested. However, *haf* transporters 1, 2 and 3 were over-expressed in an IVM resistant isolate (Yan et al., 2012). Thus, it is possible that *haf* transporters could be involved in anthelmintic resistance.

Most of the tested genes showed strong intragroup variation associated with samples from different hosts. This is expected as it is known that in sheep and goats drug exposure varies among different individual animals (Scott et al., 1990; Cerkvenik et al., 2002). Overall, selection for resistance led to upregulation only in the *Hco-pgp-9a*, *Hco-pgp-11* and *Hco-haf-9* genes. In terms of exposure, no significant upregulations in expression were observed in any of the genes studied. The high expression of *pgp*s resulting from the selection process may imply that a greater amount of ABC transporters are available in the cells and this would assist in the survival of the parasite once exposed to the anthelmintic. On the other hand, we only studied one time point and it is possible that further changes in gene expression could occur as time passes as previously described for some PGP genes in *H. contortus* (Maté et al., 2018). 

In conclusion, we showed that different ABC transporters may present expression variations in both selection and exposure scenarios consistent with the literature in general. While *Hco-pgp-9a* is generally well-studied*, Hco-pgp-11* and *Hco-haf-9* are interesting additional candidates for further studies and the search for inhibitors. ABC transporter studies offer an opportunity to develop control strategies against nematodes as the inhibition of transporter activity may be carried out using multidrug resistance inhibitors in combination with commercial anthelmintics such as macrocyclic lactones. This combined use can lead to higher exposure of the parasite to the anthelmintic and increased drug efficacy (Raza et al., 2023). While the interaction of PGPs and benzimidazoles has been studied previously and the specific interaction of oxfendazole and PGPs being rather unlikely, it cannot be totally excluded due to the expression results for *Hco-pgp-9a* obtained in this study. The wide variety of binding sites present in these proteins and that selection for benzimidazole resistance in *H. contortus* resulted in increased allelic frequencies for specific PGP allelic variants. Finally, while the interaction of IVM and PGPs is well documented, the wide variation found in our results, and other studies, regarding parasites obtained from different host animals points toward the importance of the host-parasite relationship which should be the subject of future studies. The positive regulation of cellular efflux mechanisms such as PGPs is one of the mechanisms of anthelmintic resistance and understanding how they function is important to managing this problem. Viable P-glycoprotein inhibitors, once identified, may further add to the toolbox available to mitigate the consequences of anthelmintic resistance together with other known resistance management strategies.

## Supplementary material

The following online material is available for this article:

Figure S1 - Amplified RT-qPCR products and their respective negative controls after electrophoresis on 1.5% agarose gel stained with ethidium bromide and visualized under ultraviolet (UV) light. 100 bp and 1 kb lanes: 100 bp and 1 kb Plus molecular markers (Invitrogen, Carlsbad, CA, USA); Lanes 1-6: *Hco-ftt-2* (113 bp); Lanes 7-12: *Hco-gapdh* (163 bp); Lanes 13-18: *Hco-prl9* (115 bp); Lanes 19-24: *Hco-act* (100 bp).

Figure S2 - Amplified RT-qPCR products and their respective negative controls after electrophoresis on 1.5% agarose gel stained with ethidium bromide and visualized under ultraviolet (UV) light. 100 bp and 1 kb Lanes: 100 bp and 1 kb Plus molecular markers (Invitrogen, Carlsbad, CA, USA); Lanes 1-6: *Hco-pgp-1* (128 bp); Lanes 7-12: *Hco-pgp-2* (172 bp); Lanes 13-18: *Hco-pgp-10* (188 bp); Lanes 19-24: *Hco-haf-9* (170 bp).

Figure S3 - Amplified RT-qPCR products and their respective negative controls after electrophoresis on 1.5% agarose gel stained with ethidium bromide and viewed under ultraviolet (UV) light. 1 kb Lanes: 1 kb Plus molecular marker (Invitrogen, Carlsbad, CA, USA); Lanes 1-6: *Hco-pgp-3* (107 bp); Lanes 7-12: *Hco-pgp-9a* (197 bp); Lanes 13-18: *Hco-pgp-10* (188 bp); Lanes 19-24: *Hco-pgp-11* (166 bp).

Figure S4 - Amplified RT-qPCR products and their respective negative controls after electrophoresis on 1.5% agarose gel stained with ethidium bromide and visualized under ultraviolet (UV) light. 100 bp and 1 kb Lanes: 100 bp and 1 kb Plus molecular markers (Invitrogen, Carlsbad, CA, USA); Lanes 1-6: *Hco-pgp-9b* (175 bp); Lanes 7-12: *Hco-pgp-10* (188 bp); Lanes 13-18: *Hco-pgp-11* (166 bp); Lanes 19-24: *Hco-pgp-16* (163 bp).

Figure S5 - Amplified RT-qPCR products and their respective negative controls after electrophoresis on 1.5% agarose gel stained with ethidium bromide and visualized under ultraviolet (UV) light. 1 kb Lanes: 1 kb Plus molecular marker (Invitrogen, Carlsbad, CA, USA); Lanes 1-6: *Hco-pgp-10* (188 bp).

Table S1 - Studied genes, GenBank access numbers and access numbers in the *H. contortus* genome.
