# Supplementary material for: Expression of transporter genes in anthelmintic resistant isolates of Haemonchus contortus
Source: Genet Mol Biol. 2024 Aug 16;47(3):e20230350. doi: 10.1590/1678-4685-GMB-2023-0350 (PMC11331566; doi:10.1590/1678-4685-GMB-2023-0350)
Supplement: Figure S1 - [file 1415-4757-GMB-47-03-e20230350-s1.pdf]

Genetics and Molecular Biology

https://doi.org/10.1590/1678-4685-GMB-2023-0350

Supplementary Material to "Expression of transporter genes in anthelmintic resistant isolates of *Haemonchus contortus*"

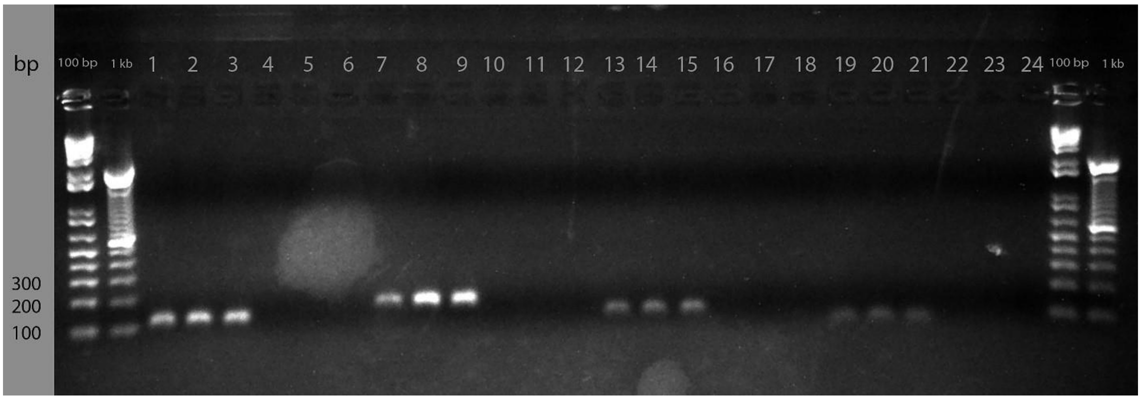

Figure S1 - Amplified RT-qPCR products and their respective negative controls after electrophoresis on 1.5% agarose gel stained with ethidium bromide and visualized under ultraviolet (UV) light. 100 bp and 1 kb lanes: 100 bp and 1 kb Plus molecular markers (Invitrogen, Carlsbad, CA, USA); Lanes 1-6: *Hco-ftt-2* (113 bp); Lanes 7-12: *Hco-gapdh* (163 bp); Lanes 13-18: *Hco-prl9* (115 bp); Lanes 19-24: *Hco-act* (100 bp).
