# Supplementary material for: Expression of transporter genes in anthelmintic resistant isolates of Haemonchus contortus
Source: Genet Mol Biol. 2024 Aug 16;47(3):e20230350. doi: 10.1590/1678-4685-GMB-2023-0350 (PMC11331566; doi:10.1590/1678-4685-GMB-2023-0350)
Supplement: Figure S3 - [file 1415-4757-GMB-47-03-e20230350-s3.pdf]

Genetics and Molecular Biology

https://doi.org/10.1590/1678-4685-GMB-2023-0350

Supplementary Material to "Expression of transporter genes in anthelmintic resistant isolates of *Haemonchus contortus*"

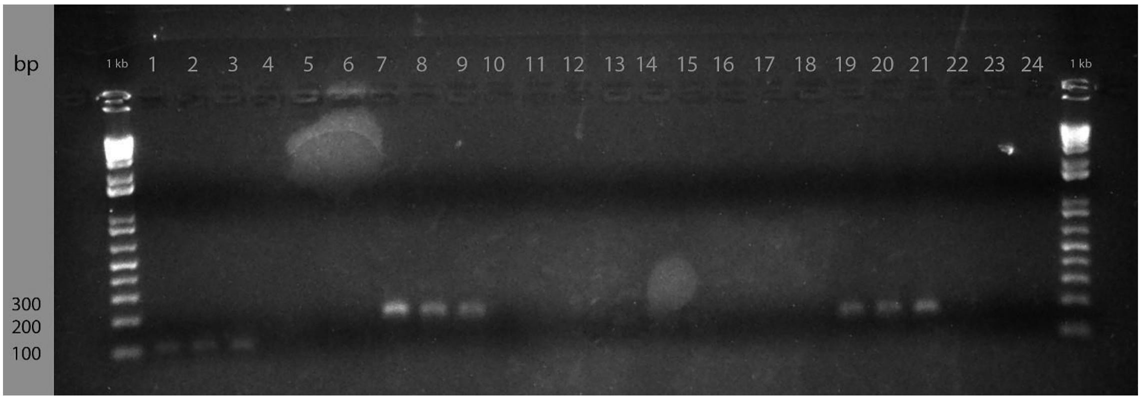

Figure S3 - Amplified RT-qPCR products and their respective negative controls after electrophoresis on 1.5% agarose gel stained with ethidium bromide and viewed under ultraviolet (UV) light. 1 kb Lanes: 1 kb Plus molecular marker (Invitrogen, Carlsbad, CA, USA); Lanes 1-6: *Hco-pgp-3* (107 bp); Lanes 7-12: *Hco-pgp-9a* (197 bp); Lanes 13-18: *Hco-pgp-10* (188 bp); Lanes 19-24: *Hco-pgp-11* (166 bp).
