# Supplementary material for: Expression of transporter genes in anthelmintic resistant isolates of Haemonchus contortus
Source: Genet Mol Biol. 2024 Aug 16;47(3):e20230350. doi: 10.1590/1678-4685-GMB-2023-0350 (PMC11331566; doi:10.1590/1678-4685-GMB-2023-0350)
Supplement: Table S1 - [file 1415-4757-GMB-47-03-e20230350-s6.pdf]

Genetics and Molecular Biology

https://doi.org/10.1590/1678-4685-GMB-2023-0350

Supplementary Material to "Expression of transporter genes in anthelmintic resistant isolates of *Haemonchus contortus*"

Table S1 - Studied genes, Genbank access numbers and access numbers in the *H. contortus* genome.

Gene	Accession number (Genbank)	Accession number (WormBase *H. contortus*)
Hco-pgp-2	AF003908.1	HCON_00004450
Hco-pgp-3	HM635768.1	HCON_00042800
Hco-pgp-9a	JX430937.1	HCON_00130050
Hco-pgp-10	HM635769.1	HCON_00168800
Hco-pgp-11	HM635770.1	HCON_00162780
Hco-pgp-16	JX430941.1	HCON_00035895
Hco-haf-9	NM_059353.4	HCON_00003100
Hco-gapdh	HM145749.2	HCON_00063590
Hco-act	DQ080917.1	HCON_00185450
Hco-prl9	Y13428.1	HCON_00084440
Hco-fft-2	NM_001267887.1	HCON_00174840
